# Naturally Occurring Terpenes: A Promising Class of Organic Molecules to Address Influenza Pandemics

**DOI:** 10.1007/s13659-021-00306-z

**Published:** 2021-05-03

**Authors:** Suhad A. A. Al-Salihi, Fabrizio Alberti

**Affiliations:** 1 Applied Sciences Department, University of Technology, Baghdad, Iraq; 2 School of Life Sciences and Department of Chemistry, University of Warwick, Gibbet Hill Road, Coventry, CV4 7AL UK

**Keywords:** Influenza A, Virus, Fungal terpenes, Plant terpenes, Antivirals

## Abstract

Since the olden times, infectious diseases have largely affected human existence. The newly emerged infections are excessively caused by viruses that are largely associated with mammal reservoirs. The casualties of these emergencies are significantly influenced by the way human beings interact with the reservoirs, especially the animal ones. In our review we will consider the evolutionary and the ecological scales of such infections and their consequences on the public health, with a focus on the pathogenic influenza A virus. The nutraceutical properties of fungal and plant terpene-like molecules will be linked to their ability to lessen the symptoms of viral infections and shed light on their potential use in the development of new drugs. New challenging methods in antiviral discovery will also be discussed in this review. The authors believe that pharmacognosy is the “wave of future pharmaceuticals”, as it can be continually produced and scaled up under eco-friendly requirements. Further diagnostic methods and strategies however are required to standardise those naturally occurring resources.

## Introduction

A glimpse at the preoccupation of yet to spread pathogens, may suggest unfounded safety precautions, however, the huge public health consequences of measles, malaria, tuberculosis exemplified the influence of late underpinnings of new disease [1]. Although, not all new infections have had huge health problems as the above mentioned examples, the outbreak and the devastating effect of HIV on human population, is an obvious counterargument of the possibility of novel infections impact on public health and their subsequent economic catastrophe [2].

The infection process of most emerging diseases involves two steps: introduction and adoption. Firstly, the infection agent invades a new host (regardless of its origin, environment, or different species), and secondly the agent’s establishment inside the host [3]. Although, first incidents of pandemics are thousands of years back, the emergence of new epidemics or the re-emergence of known infections, continue as the interaction between humans and animals including their niches increases. There are many reasons for global disease outbreaks, individual movements for example, can spread previously controlled infections in developed countries to others with poor health systems. Microbial adaptation to environmental changes, such as developing resistance to antibiotics—which is a consequence of antibiotics overuse in animal farming and food processing—increases the chances of the emergence of new strains of pathogens. Ecological changes such as deforestation, can also increase the likelihood of indirect contact with different types of insect vectors and mammalian reservoirs. The infection of some diseases can increase during the cold season, like influenza in winter. As well as the ease in the international travel of goods, animals, and humans, made the transmission of diseases or their vectors much easier yet more difficult to control [4]. Above all, shortage of public health facilities, such as drinkable water, educational programmes and lack of living expense can participate largely in disease outbreak as well as the re-emerging of previously contained contagions [5]. Since the 1940s, the human lifespan has been greatly improved by the introduction into the drug discovery field of naturally produced fungal bioactive molecules, as well as of their semisynthetic derivatives and synthetic analogues inspired by natural products. However, nowadays we simultaneously face a challenging rise in antimicrobial resistance, and a dramatic decrease in drug innovation. The chemical constituents that are responsible for the bioactivities of fungi are mostly terpene derivatives, some of which (monoterpenes and sesquiterpenes) are volatile in their nature. These compounds are overly broad in their pharmacokinetic spectrum, and often inspired structure-based drug design, particularly in oncogenic and contagious diseases. Insightful knowledge of the chemistry, biogenetic and biotechnology associated to them, will fruitfully increase the application of their native producers—mushroom forming fungi [6].

It is therefore important to better our understanding of the geographical and natural evolution of infectious diseases, as well as of the bioactive metabolites made by living organisms such as mushroom-forming fungi, to develop new tools to defeat future microbial threats, through effective coordinated global interactions.

## Influenza Viruses

### Evolution of Influenza Viruses

**Fig. 1:**
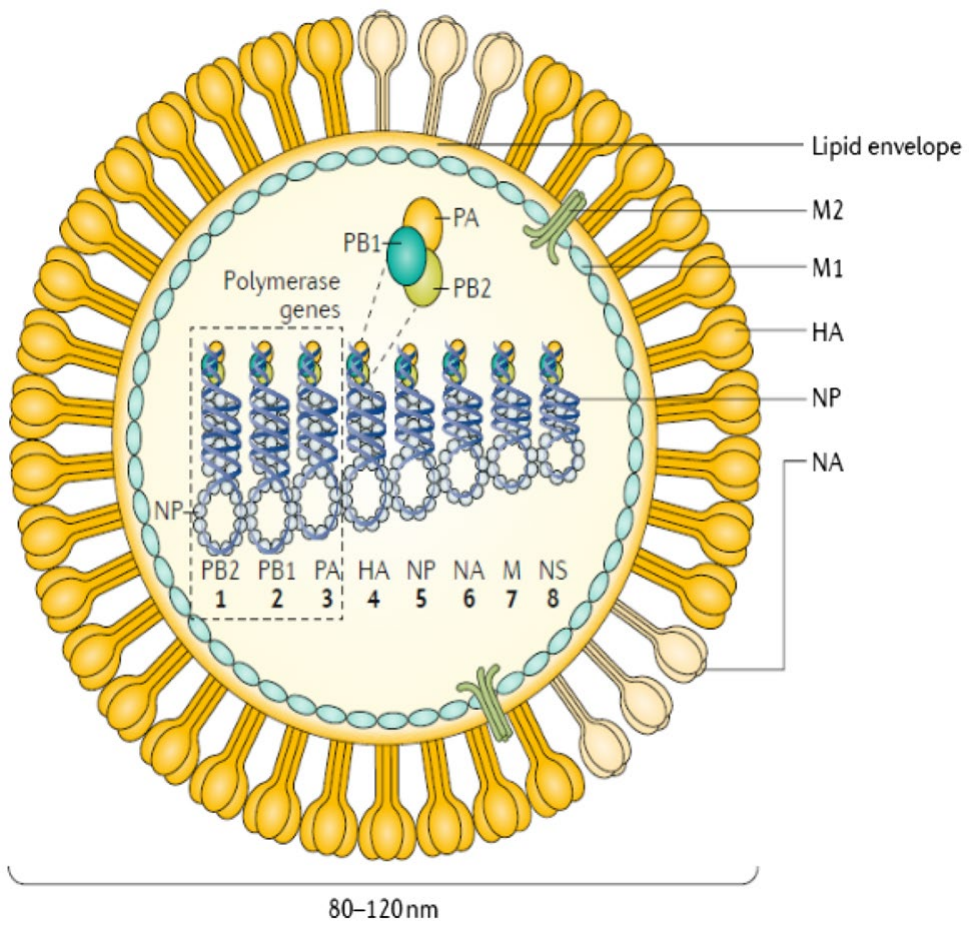
Structure of influenza A virus. The viral genome consists of eight RNA connected genes, surrounded by the lipid envelope, to which the neuraminidase and hemagglutinin proteins are attached in addition to the matrix and the membrane proteins

Influenza viruses are a group of microbes belonging to the family *Orthomyxoviridae*. They are negative single stranded RNA viruses, causing severe emerging and re-emerging respiratory infections in human, due to their ability to alter their genomes continuously (Fig. 1).

**Fig. 2:**
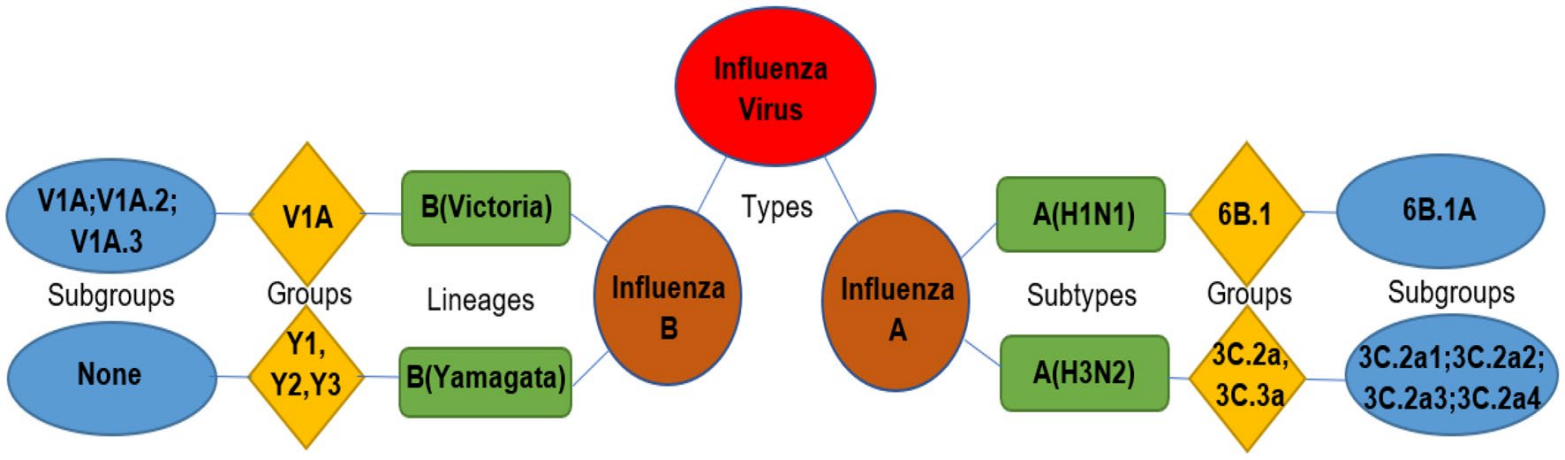
Main two causes of human seasonal flu; influenza viruses A & B. These are further classified into groups and subgroups

They are classified into four main types: Alpha influenza (A), Beta influenza (B), Gamma influenza (C) and Delta influenza (D) [7]. A and B are known as seasonal viruses or the so called “human influenza”, they mainly cause seasonal flu epidemics, yet influenza type A is the only one that has caused influenza pandemics to date—epidemics develop to pandemics when new strains of microbes infect people and can spread globally. While type C is responsible for mild symptoms in human, with limited spread ability, type D mainly infect cattle [8]. According to proteins Neuraminidase (N) and hemagglutinin (H) that are attached to their surface, viruses can be divided into many subgroups. N includes 11 subtypes and H includes 18 subtypes. Nearly 198 subtypes of influenza virus A have been predicted, of which 131 subtypes have been confirmed. Types that frequently infect people are, influenza A(H1N1) and influenza A(H3N2). The latter has the tendency to change its genetic material rapidly. Like type A, influenza type B is classified into two subgroups; Yamagata and Victoria which are further divided into many subclades (Fig. 2). Cases of co-circulation of both subgroups were reported around the world, however, with variable geographical distribution of each subgroup. Generally, the genetic material of type B is more stable than type A in terms of rate of changes/mutations [9].

The annual infection rates of influenza A and B in humans range from 5 to 15%, of which children represent the higher percentage (nearly a third) of infection incidences. Since the human immune system cannot save the memory of previous influenza viruses for a lifetime, individuals are at risk of recurrent infections. This reflects the fact that the immune system of every adult has a history of viral infections [10].

The first detection of influenza viruses dates back to the sixteenth century, during the Russian influenza pandemic. Generally, influenza viruses transmit from their primitive hosts to their intermediate ones to ensure better evolution and subsequent transmission. Not long ago, the infection landscape of influenza has expanded widely to include previously unhabituated hosts such as whales, seals, and bats. Influenza A for example, affects a wide range of mammalians including pigs, dogs, seals, cats and humans, where the latter become an intermediate host for numerous viruses [11]. Viral infections largely take two routes: the first one is the isolated route or the unsustainable infection, where the microbe fails to adapt or evolve in its new host such as Hantavirus Pulmonary Syndrome and Ebola disease. In the second route the virus (e.g. influenza) adapts in the intermediate host, and successfully transmits to a new environment, leading to durable establishment that can resist any harsh environment posed by the end host [4]. There are many factors—viral and host—that influence an effective transmission and sustainability of any infection. Viral factors include the ability to produce adaptive mutation proteins, such as HA glycoprotein, PB2, NP and NS1 proteins. Other viral factors are its interconnectivity and population size. Host determinant factors represented by the presence of target receptors or the availability of innate immune response factors [12]. Avian influenza polymerase protein for example, has a limited influence in human infections, therefore, host-based genetic mutation occurred within its units as a result of natural evolution, leading to successful adaptation in human host [13].

### Pathogenic Properties of Influenza Viruses

Almost all human respiratory infections come from influenza viruses, which have been around since the middle ages. They infect infants, people with chronic diseases, causing them high illness and demise. For example, in 1957 in a seasonal epidemic of influenza A, nearly two hundred thousand Americans were hospitalized, of which 15% died [14]. Historically, every decade, influenza viruses’ pandemics emerge to infect more than half of the world population of which 1–2% die. On the other hand, influenza B and C, cause periodic epidemics and endemics with no pandemic records to date [7]. In terms of pathogenicity, influenza A, has the riskiest consequences on human health. However, associated health conditions (bacterial pneumonias) of infected population, seems to vary from infections to infections. The 1918 influenza pandemic for example, has caused high fatality frequency in young adults compared to elderly people, contrasting the two previous pandemics, where high mortality rate occurred in elder people and cases with chronic disease history [15]. The recent outbreak of coronavirus (COVID-19) makes revising previous influenza pandemics valuable. Largely, influenza viruses adapt to new environments via changing their surface proteins. These changes are either accumulative or straightway. The former occurs due to limited mutations in hemagglutinin (HA) and neuraminidase (NA) antigens [16]. Accumulative changes happen every time the virus replicates, and normally produce types that are closely related to the original one, therefore they have analogous antigenic properties, that trigger similar response from the host immune system, resulting in cross protection. However, over time this process can result in strains that are antigenically diverse. A small mutation in one important domain that has a huge impact on virus antigenic properties, can lead to antigenic deviation. This will change the virus to the point that the body’s immune system cannot recognize these newer antigens, and ultimately become susceptible to infection again. This is one of the main reasons why one can be infected with influenza virus many times [17]. On the other hand, straightway changes or shift changes that cause the creation of novel NA and HA proteins, resulting in newer types of influenza A. Such shifts happen when viruses from different hosts (e.g. mammalians) acquire new virulence ability to infect the human body, which has no immune defence against such virus types due to differences in their antigenic proteins. The H1N1 virus pandemic in 2009 is an example of this sort of shift, when it moved from its original host swine to humans. However, such intraspecies changes are less frequent compared to the interspecies ones [18, 19].

However, unfortunately, very few analyses concerning fatal cases of H5N1 have been published so far. One study demonstrated that the fatality of H5N1 is due to the unique hypercytokinemia, another study proposed the ability of H5N1 to replicate outside the respiratory paths [20]. However, the possibility of other pathological reasons remains unclear.

## The Potential Role of Terpenoid Natural Products in Viral Infection Treatment

Although recent flu epidemics were less incurable compared to past epidemics, there is still an urgent need to search for new approaches to prevent flu pandemics, especially the ones caused by the influenza A. Unlike bacteria, viruses cannot reproduce in the absence of a living host. Once they are inside the host cell, they hijack its replication machinery, and some of them start replicating, then they eventually burst open the cell and infect more host cells [21]. Targeting the virus replication is easy, meanwhile, avoiding the disruption of the host cell replication is the hard process. One effective way would be the synthesis of multitarget molecules, that can inhibit multiple phases of the virus life cycle [16].

Volatile natural products have shown their efficiency as antibacterial agents, and investigation of their antiviral activity is vital. The experimental improvement of influenza symptoms such as headache, runny nose and malaise during the co-use of volatile oils with synthetic medicines, suggests that volatile compounds can have an impact in lessening influenza consequent spread [22].

Generally, antiviral drugs have been designed to stop viruses from inserting their viral genomes into the host cell, as well as to avert the new copies from infecting other host cells. Ganciclovir and acyclovir, for example, are designed to inhibit the synthesis of the viral DNA of cytomegalovirus and simplex virus, respectively [23, 24]. However, those DNA inhibitors are not effective against viral RNA such as hepatitis and influenza viruses. Besides that, many species of influenza virus have developed resistance to the currently used medications, as such, more viral genome specific drugs are needed, to kill a specific virus, or control the spread of similar ones [25]. Aromatics or natural oils are a chemically diverse group of bioactive substances; thus, they have the potential to become novel antivirals, that can effectively interrupt virus development or block their invasion [26, 27].

### Fungal Antivirals

Over the last decades, tens of thousands of metabolites have been described from the fungal kingdom, many of which possess diverse medicinal properties including antibacterial, antifungal, antiviral, and antitumor. However, fungal natural products with antiviral properties were less lengthily investigated. Nevertheless, the number of fungal antiviral studies is gradually increasing, particularly, those that are related to medicinal mushrooms [28]. Although, many of the selected molecules in the below table (Table 1) have a wide range of bioactivities, we focused our scope on their antiviral properties. The described chemicals, belong to an array of fungal terpenes, and they were selected through an extensive literature search. 6-epi-ophiobolin K is a member of the ophiobolins group of sesterterpenoids, having a molecular formula C_25_H_36_O_3_. It is produced by a strain of a *Neosartorya* sp, and it showed HIV-1-integrase inhibitory activity [29]. 6β,9α-dihydroxy-14-*p*-nitrobenzoylcinnamolide is a rare sesquiterpenoid molecule (formula C_22_H_25_NO_8_) with antiviral activity against H3N2 and EV71, and it is produced by the marine-derived ascomycete fungus *Aspergillus ochraceus* [30]*.* Equisetin is a tetramic acid like compound isolated from *Fusarium equiseti*, with substituted aliphatic bicyclic ring, having molecular formula C_22_H_31_NO_4_ and shows inhibition activity on HIV-1 integrase [31]. Integracide A (C_32_H_50_O_8_S) and integracide D (C_36_H_58_O_8_) also show inhibition of the HIV-1 integrase [32], and are tetracyclic triterpenoids made by *Fusarium* sp. The oblongolides are tricyclic compounds derived from the fungus *Phomopsis sp*. BCC 9789 and display anti-HSV-1 activity [33]. The last two compounds derived from ascomycete fungi included in Table 1 are stachyflin, with molecular formula C_23_H_31_NO_4_, and stachybotrylactam, C_23_H_31_NO_4_, sesquiterpene derivatives isolated from the *Stachybotrys sp*. that exhibit inhibition on HSV-1, H1N1 and H2N2 [34–36]. The table also includes molecules characterised from basidiomycete species such as the sesquiterpene Illudin S, C_15_H_20_O_4_, which is a highly reactive molecule that can bind to the DNA and cause its damage and was first isolated from the Jack o’ Lantern mushroom *Omphalotus illudens*, showing inhibition of HSV-1 [37]*.* The bis-sesquiterpene agrocybone, molecular formula C_30_H_36_O_5_, has an eight-ring structure derived from the binding of two molecules of illudane via Diels–Alder reaction and shows weak antiviral properties against the respiratory syncytial virus (RSV) [38]. Triterpenoids with antiviral activity have been abundantly described as being isolated from the fruiting bodies of *Ganoderma lucidum* mushrooms*,* such as the lanosterol derived triterpenoid ganoderic acid Y, molecular formula C_30_H_46_O_3_, as well as many closely related triterpenoids, including the oxidized lanostane triterpenoids lucidenic acid P, C_29_H_44_O_7_, lucidumol B, C_30_H_50_O_3_, and ganolucidic acid A, C_30_H_44_O_6_ [39–41]. *Ganoderma colossum* produces the lanostane triterpenes schisanlactone A, formula C_30_H_40_O_4_, colossolactone E, C_32_H_54_O_6_, and colossolactone V, C_35_H_54_O_9_, which show anti-HIV-1 protease activity [42]. Another species of the *Ganoderma* genus, *Ganoderma pfeifferi*, produces antiviral triterpenoids such as lucidadiol, C_30_H_48_O_3_, ganoderone A, with molecular formula C_30_H_46_O_3_, applanoxidic acid G, C_30_H_40_O_8_, and lucialdehyde B, C_30_H_44_O_3_ [43, 44].

**Table 1:** Species of fungi that produce terpenes with antiviral activities

Fungal species	Used technique	Test influenza	Mechanism of action	References
	In vitro	HIV-1	Inhibits the strand transfer reaction of HIV-1 integrase	[29]
	In vitro	H3N2 and EV71	Inhibitory activity	[30]
	In vitro	HIV-1	Inhibits HIV-1 integrase	[31]
	In vitro	HIV-1	Interferes with the replication of HIV-1	[32]
	In vitro	HSV-1	Antiviral activity	[33]
		HSV-1, H1N1 and H2N2	Inhibits the HA-mediated virus-cell fusion process	[34–36]
	In vitro	HSV-I	Antiviral activity	[37]
	In vivo & in vitro	RSV	Antiviral activity	[38]
	In vitro	EV71, HIV-1 and EBV-EA	Blocks EV71 uncoating to inhibit viral replication, Inhibits HIV-1 protease and inhibits EBV-EA induction	[39–41]
	In vitro	HIV-1	Inhibits HIV-1 protease	[42]
	In vitro	H1N1and HSV-1 HSV-I	Antiviral activity	[43, 44]

Bioactive compounds are organic molecules with normally small molecular weight that can be produced by both Ascomycetes and Basidiomycetes. Literature reports on fungal antiviral sources reveal that most known bioactive molecules have been isolated from Ascomycetes and Basidiomycetes, providing insights into the competence of those two phyla as producers of therapeutic compounds [45–48]. The studies indicated also that the biosynthetic gene clusters of the bioactive metabolites’ dominance are generally lower in number in basidiomycetes compared to the ascomycetes. However, this difference reflects the bias from the use of different techniques and tools in isolating and characterising the bioactive compounds, and not their genuine productivity of biologically active metabolites [49]. Current reports on fungal diversity prediction, suggested that less than 10% of fungal species are described, which again demonstrates the great potential of fungi as a source of antivirals [50]. Above that, medicinal mushrooms played an important role in natural remedies, their healing power has been subjected to vast scientific research, such as the edible mushroom shiitake. Many other species of mushroom-forming fungi exhibited potential to produce natural antivirals. Reports on mushrooms bioactivities, linked their ability in defeating viral infections to the presence of two types of chemicals: polysaccharides and terpene like compounds [51]. However, other types of biologically important molecules have been reported from mushrooms, implying the potential content of structurally diverse biomolecules that are awaiting discovery [52]. Historically, terpene derivatives are one of the most widespread naturally occurring products, that are predominantly found in the form of aromatic oils. Such products were mainly used, for instance, for religious reasons in the olden Egypt. Camphor (a terpene isolated from essential oils of camphor tree) for example, was first introduced to Europe by Arabian traders ten centuries ago. In 1818 researchers were able to analyse turpentine oils and then propose the term “terpene” instead of camphor, which was used to describe extracted crystalline oxygenated molecules from essential oils. Further analysis by other researchers has resulted in the description of the building block “isoprenic” of this molecule [53]. The role of mevalonic acid in cholesterol biosynthesis and its incorporation in the synthesis of many terpene compounds was defined in 1956. Following this, thousands of terpenoids were structurally and functionally characterised. Apart from their antimicrobial activities, terpenes have also been reported as hormones and photosynthetic pigments [54].

### Aromatic Natural Oils

Research on essential oils extracted from plants, demonstrated the dominance of terpene-like compounds. particularly monoterpenes and triterpenes, as they represented more than 95% of the chemical constituents of those oils [55]. We, therefore, further searched for promising antiviral terpenes characterised in plants and presented them in Table 2. These include eugenol (molecular formula C_10_H_12_O_2_), a volatile phenolic compound with antiviral properties, which represents an essential constituent of clove oil [56, 57]. Germacrone, formula C_15_H_22_O, is the sesquiterpene constituent of the essential oil of many globally distributed plants and shows inhibition activity on the influenza virus [58, 59]. Patchouol, formula C_15_H_26_O, is a sesquiterpene alcohol mainly found in patchouli plant leaves, and has shown promising results as an inhibitor of influenza viruses [59–62]. Interestingly, this compound is also used as a precursor in the synthesis of the anticancer compound taxol. β-santalol, formula C_15_H_24_O, is another example of a sesquiterpene type compound, found in *Santalum album*, in which it represents one fifth of the total plant essential oil [63]. Terpinen-4-ol and α‐terpineol, both with chemical formula C_10_H_18_O, are components of the essential oil of the tea tree and many other aromatic plants, and they show antiviral activity [64–66]. Terpinolene belongs to the group of monoterpenes, with chemical formula C_10_H_16_, and can be naturally sourced from different plants, such as cardamom.

**Table 2:** Examples of some terpenes that are primarily produced by plants and their antiviral activity and mechanism of action

Volatile terpen compound	Used technique	Test influenza strain	Mechanism of action	References
	In vitro	H1N1- 4 strains, H3N2-2 strains, H9N2-2 strains, H5N1-1 strain	1. Disrupts the viral reproduction and the subsequent hypercytokinemia 2. Inhibition or reduction in the interaction of NA and HA proteins 3. Prolongs cell proliferation	[56, 57]
	In vitro*,* in vivo	H1N1-2 strains, H3N2-2 strains, Influenza B virus-1	Interferes with the transcription machinery and attachment of the virus proteins prior and during the infectious process	[58, 59]
	In vitro*,* in vivo	H1N1-2 strains, H2N2-1 strain, H3N2-1strain, Influenza B virus-1 strain	1. Disrupts the viral reproduction and the subsequent hypercytokinemia 2. Inhibition or reduction in the interaction of NA and HA proteins	[59–62]
	In vitro	H3N2-1 strain, H1N1-1 strain	Interacts with the translation process of the viral genome to reduce the amount of the produced mRNA	[59, 63]
	In vitro	H1N1-1 strain	Causes reduction in cell transportation networking during the acidification process	[64–66]
	In vitro	H1N1-1 strain	Causes reduction in cell transportation networking during the acidification process	[65, 66]
	In vitro	H1N1- 1 strain	Causes reduction in cell transportation networking during the acidification process	[65, 66]

Aromatic natural oils are bio-generated via differentiated pathways in plants, fungi as well as some species of bacteria. Their main components belong to the highly variable family of natural products—terpenes—including derivatives of ketones, alcohols, phenols, esters and aldehydes. Over the last decade, volatile chemicals regained interest as sources of new antimicrobials and anticancer compounds. Although, they were mainly used against carcinogenic and bacterial infections, their potential anti-inflammatory, antioxidant and immunomodulatory are reasonably investigated [56, 67].

Essential oils contain many functioning compounds, including mono- and sesquiterpenes. Many of those volatile substances can be competently used in the medication of different types of cancers, either as antitumor or supportive compounds [68]. The awareness of terpene derived compounds as potential antitumors started when clinical researches showed their distinguished biological activity and their non-toxic impact on human’s normal cells, as many of them have been stated as Generally Regarded As Safe (GRAS) substances, which represents key criteria for their use for antibiotic discovery [69].

Terpenes are a large class of natural metabolites that comprise over two thirds of all known secondary metabolites. They are biosynthesised by most living organisms including plants, fungi and bacteria, however many of these compounds investigated by scientists are from plants or mushroom-forming fungi [70]. Compared to the current knowledge of chemicals produced by fungi, little is known about their synthesis and function—especially for terpenes. The antagonistic or synergistic interactions of those natural substances with other chemical or biological elements, can negatively influence their therapeutic properties. It is, therefore, important to use well-defined molecules in terms of organism, enzymes, biosynthesis routes and activity. Since those biologically interesting terpenes are produced in small quantities by their native organisms, there is a reasonable need to thoroughly investigate their synthesis via combinatorial pathways—as classical chemical synthesis proved ineffective in terms of purity and environmental impact of catalysts—and in the presence of selective enzymes and affordable simple terpenes, such as pinene and limonene [43, 71–73].

### Biotechnological Strategies for Drugs Synthesis

Normally, terpene biosynthetic genes are customarily located next to each other in one biosynthetic gene cluster, easing their exploitation and manipulation to produce bioactive compounds with desired quality and quantity [74]. Historically, more than half of the drugs in use, are sourced from natural substances or their derivatives. With the recent advancements in genomic analysis and analytical tools, new strategies and methods have been utilised for the exploitation of naturally synthesised compounds that are of biotechnological importance [75]. The main aim of using such techniques was to predict the genes encoding molecules with novel bioactivity. These techniques have further established the fact that the capability of mushroom forming fungi to synthesise bioactive molecules has been overlooked. They have also revealed the presence of the so called “cryptic” or “orphan” biosynthetic gene clusters [76]. The pressing need to produce new antibiotics due to the non-stop increase in antimicrobial resistance and the decline in efficiency of accessible drugs, highlights the importance of using unprecedented techniques such as genome mining in drug discovery. This approach can simplify the biosynthesis of biologically engineered compounds via reducing their commercial production and use [77]. This genome mining method can be applied in many different ways. One way is the detection of compound’s coding genes, through “target directed genome mining”, which mainly consists in the identification of biosynthetic gene clusters with unknown function in terms of the corresponding molecule(s) produced [78]. Another way is “the one strain many compounds approach”, this method can be used to induce the expression of cryptic genes through controlled alteration of growth conditions. Many silent metabolic pathways were activated using such technique, including activation of the biosynthesis of polypeptides and polyketides with pharmaceutical applications [79, 80]. Metabolism regulation can also be manipulated to enhance the production of secondary metabolites with novel bioactivity. These treatments involve the use of chemicals or small organic molecules to modify or deregulate metabolic processes through the inhibition of fatty acids [81]. Examples of other techniques used concurrently with genome mining-based studies are the heterologous expression of biosynthetic gene clusters that synthesise known bioactive molecules. These reconstructed routes ensure the production of pure and easily identifiable intermediates and eliminate unusable by-products. Despite that many terpenes with antimicrobial activity have been described, many more with potential unique derivatives are awaiting discovery [82, 83].

## Conclusions

Even though influenza viruses are of relatively old origins and very contagious, the currently used antivirals are undoubtedly limited in terms of their bioactivity spectrum and sources. What makes things even more concerning and further intensifies the need for new strategies in virucide discovery, is the constant increase in antimicrobial resistance. Typical antibiotic discovery was and still involves the isolation, characterisation, and testing methods against panels of pathogenic bacteria, without considering the presence of other life-threatening germs, which are viruses in this case. Lately, several literature reports described the antiviral activity of a variety of volatile compounds against some viruses including influenza. We therefore attempted to provide a brief outline on influenza virus structure, evolution, pathogenicity and then suggest terpenes as potential sources to develop antivirals. Our literature search showed that viruses can be virulent and destructive in many levels and difficult to control unless urgent innovative policy is made in the drug discovery field. It also highlighted terpenes as potential antivirals due to the natural sustainability they demonstrated when tested against many viruses.

The recent advances in genetic tools, molecules structure modelling, and large-scale screening, established the guidelines for the discovery of novel antibiotics against infectious diseases. Current strategies involve, but are not limited to, two approaches. One is to characterise or improve drugs against known microbes. Ideally this method can be expanded to involve different types of viruses including the drug resistant ones. Second, is the validation of new targets in both the host and the virus, to accordingly develop novel classes of molecules, via either structure-based molecule interaction, or host transport machineries. The viral polymerase, for example, is an internal protein that can be further exploited according to its structure interaction with new molecules. Targeting the transportation system of the host cell, it can also provide a new avenue for virucide development, especially for viral RNPs, however, potential intrusion with the metabolic system of the host should be considered.

Undoubtedly, current computational genome sequencing, have effectively pushed the medical search generally and infectious disease search particularly, towards further understanding of the interaction between the microbial or viral communities and the human body, and determine their virulence or symbiosis at the molecular level. However, despite this progress in the genomic era, infectious diseases still cause high frequency of illness and mortality among populations, especially in the developing countries. It is therefore vital for those countries to strengthen the input of bioinformatics in such field of research, through more well-designed training, internship courses and collaboration with more developed specialised institutions around the globe, to help to control the spread of contagious diseases worldwide.

Another way that bioinformatics has contributed to the field of infectious diseases, was the development of a new era of bioactive natural products research, through linking many bioactive natural products to their associated biosynthetic gene clusters and corresponding enzymes. Although this approach has proven to be effective in identifying bioactive molecules and the BGCs that are associated to them, they are still in their infancy and represent a new shift in sustainable drug discovery, especially for plants and animals.
